# Supplementary figures and images for: T Cell-Macrophage Interactions Influence Chemotherapeutic Response in Ovarian Cancer Patients
Source: Int J Mol Sci. 2026 Jul 10;27(14):6176. doi: 10.3390/ijms27146176 (PMC13409878; doi:10.3390/ijms27146176)

# Supplementary figure S1

a

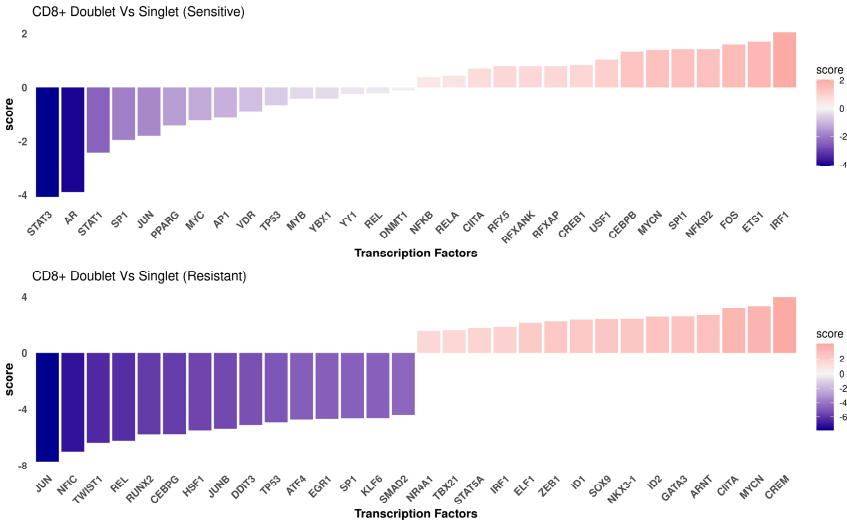

b

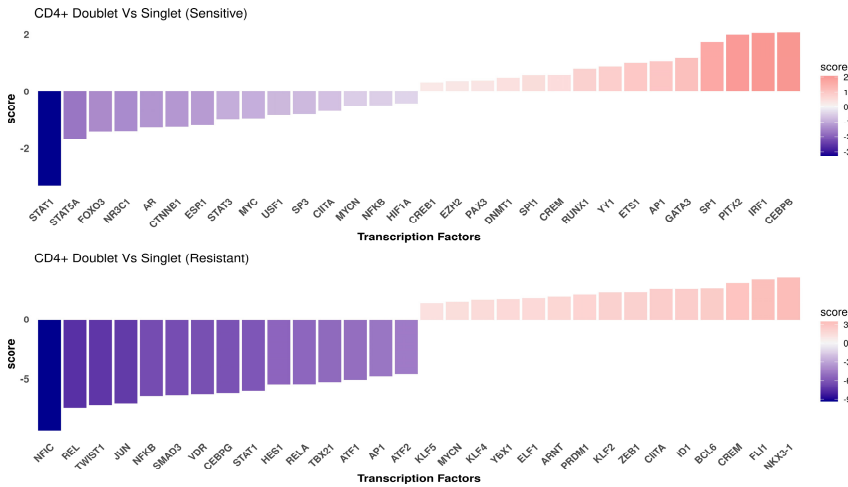

Supplement: Supplementary file 1 [file ijms-27-06176-s001.zip › Supplementary Figure S1.pdf]
